# Interleukin-22 participates in the inflammatory process of vitiligo

**DOI:** 10.18632/oncotarget.22644

**Published:** 2017-11-24

**Authors:** Jinjin Dong, Xiaohong An, Hui Zhong, Yichuan Wang, Jing Shang, Jia Zhou

**Affiliations:** ^1^ School of Traditional Chinese Pharmacy, China Pharmaceutical University, NanJing 2111198, P.R. China; ^2^ Jiangsu Key Laboratory of TCM Evaluation and Translational Research, China Pharmaceutical University, Nanjing 211198, P.R. China; ^3^ State Key Laboratory of Natural Medicines, China Pharmaceutical University, NanJing 210009, P.R. China

**Keywords:** IL-22, IL-1β, NLRP3, vitiligo, inflammatory process

## Abstract

Vitiligo is an acquired depigmentary skin inflammatory disorder. The pathogenesis of inflammatory skin disease involves the release of cytokines from keratinocytes, including interleukin (IL)-1β. IL-22 belongs to a family of cytokines structurally related to IL-10, including IL-19, IL-20, IL-24, and IL-26. In contrast to IL-10, IL-22 has proinflammatory activities. Among skin cell populations only keratinocytes are the major targets of IL-22. In the present study, we demonstrated that IL-22 promoting IL-1β secretion from keratinocytes via the Reactive oxygen species (ROS)-NOD-like receptor family, pyrin domain containing 3 (NLRP3)-caspase-1 pathway. It inhibited the expression of protease-activated receptor-2 (PAR-2) of keratinocytes. However, IL-22 had no direct effect on normal human foreskin-derived epidermal melanocytes (NHEM). Considering the closely connection between keratinocytes and melanocytes, and the ability of keratinocytes to produce a plethora of cytokines, in the present work, we examined whether IL-22 could regulate melanocytes functions by keratinocytes participation. Keratinocytes were exposed to IL-22 and the conditional medium was collected. The effect of conditional medium on melanocytes was studied. The expressions of relative proteins were assessed by western blot. Influence of conditional medium on NHEM migration was assessed by Transwell method and the apoptosis by flow cytometry analysis. The IL-22-treating keratinocytes conditional medium inhibited melanogenesis and restrained the expressions of Rab GTPases of NHEM. In addition, the conditional medium suppressed melanocytes migration and induced apoptosis. Our results collectively indicated that IL-22 may potentiate IL-1β-mediated skin inflammation and result in participating in the inflammatory pathogenesis of vitiligo.

## INTRODUCTION

Skin works as a bio-barrier against outside influences and also as pigmentary system [1, 2]. As playing protective functions, the skin mobilizes a large amount of cell populations to act in a coordinated fashion. Skin’s neuroimmunoendocrine axes is established by epidermal keratinocytes, melanocytes, and dendritic Langerhans cells (LCs), combining with dermal fibroblasts, macrophages, mast cells and others [3, 4]. These communication and interaction among cells are passed through the multiply kind of molecules termed cytokines. Cytokines play an important role in modulation of body homeostatic response. They are classically secreted “messenger” proteins that allow for cell-cell communication [5, 6].

Vitiligo is an intriguing depigmentary disorder affecting approximately 0.5-2% of the world population. Numerous factors have participated in the development of vitiligo, histologic evidence indicates that vitiligo is an inflammatory disease with development of a lichenoid tissue reaction [7]. It develops due to progressive disappearance of epidermal melanocytes. Melanocytes can detect and decode the solar or thermal energy, or respond to biological of physicochemical signals to sense the environment. All these functions of melanocytes promote forming a hypothesis that melanocytes are ‘neurons of the skin’ [8].

In the skin, inflammasome protein NLRP3 can be activated by UV exposure and sensitizers, which induces IL-1β production [9, 10]. IL-1β mRNA expression was elevated in the lesioned edge of vitiligo [11]. Upregulation of IL-1β transcript in patients advocates its possible role in autoimmune pathogenesis of vitiligo [12]. When co-cultured with activated T cells, IL-1β secretion and caspase-1 expression was increased of keratinocytes [13]. IL-22 belongs to IL-10 family, which plays a vital role in various inflammatory and infectious diseases. IL-22 is primarily produced by obvious immune cells, and Th22 cells are a major source of IL-22 in many diseases [14–16]. When treated with IL-22, keratinocytes could be triggered to induce inflammatory responses [17]. The functional heterodimeric receptor of IL-22 comprised of IL-10RB and IL-22RA1 subunit [18–20]. IL-10RB subunit is the ubiquitously expressed one while the IL-22RA1 subunit is more restricted. IL-22RA1 subunit is mainly expressed on epithelial cells, keratinocytes, and hepatocytes, but are mostly absent from cells of hematopoietic origin [21–23]. The restricted expression of IL-22RA1 defines that the target tissues of IL-22 are special correspondingly, including skin, small intestine, lung, kidney and so on [24, 25]. The downstream signaling of IL-22–IL-22R system is mediated through the Janus kinase (JAK) –signal transducer and activator of transcription (STAT) pathway, primarily activate STAT3 [26, 27].

Considering the closely connection between keratinocytes and melanocytes, and the ability of keratinocytes to produce a plethora of cytokines [28], we evaluated the influence of IL-22 on melanocytes functions. We demonstrated that IL-22 could regulate melanocytes functions by keratinocytes participation.

## RESULTS

### IL-22 induces the anti-microbial peptide and chemokines from HaCaT cells

**Figure 1:**
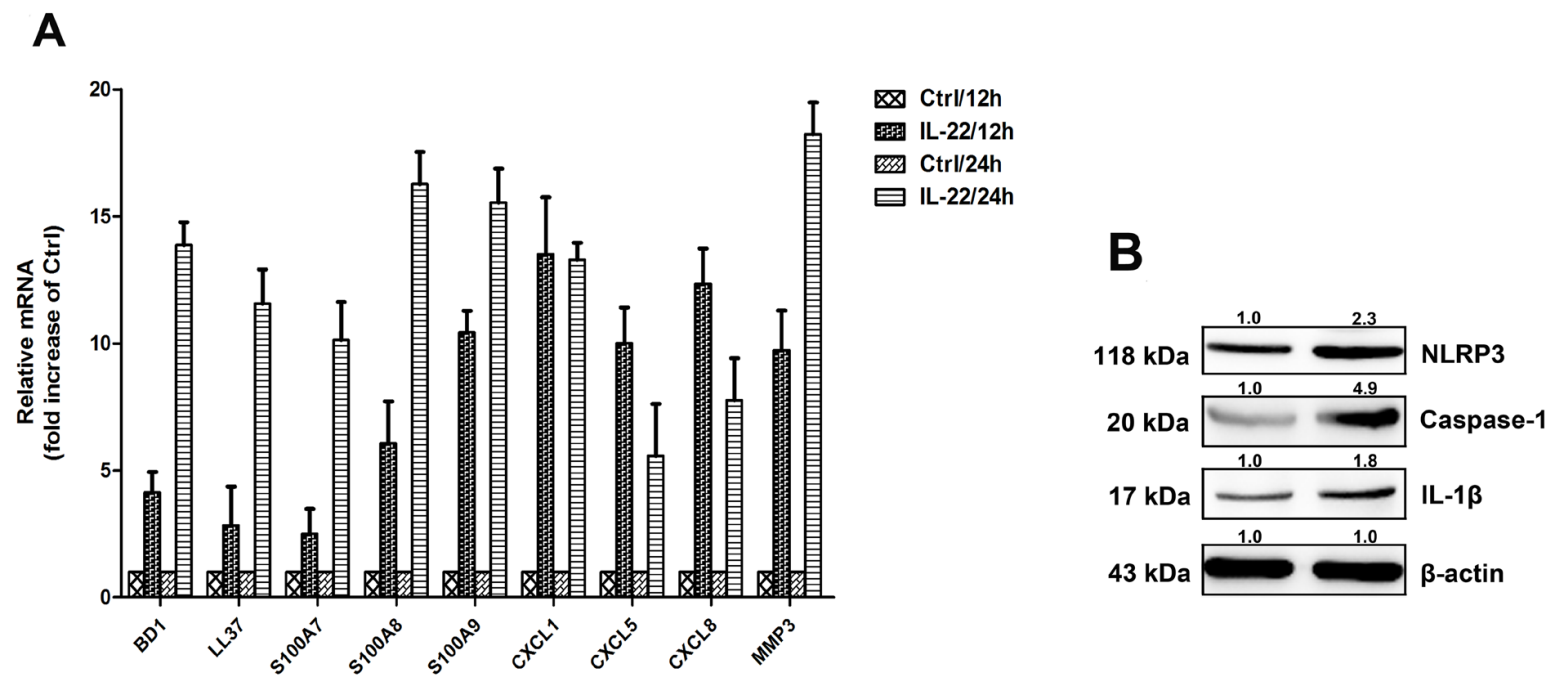
IL-22 induces the anti-microbial peptide and chemokines from HaCaT cells **(A)** HaCaT cells were treated with 20ng ml/1 of either IL-22 for 12 or days 24 hours, then the cells were harvested. The relative expressions of anti-microbial peptides and chemokines were measured via real-time RT–PCR. Data are expressed as the means ± SD (i.e. n=3) and compared with those of the ‘no treatment’ group on each of the experimental days. Data were analyzed by Two-Way Analysis of Variance (ANOVA). **(B)** Immunoassays of cell lysates from HaCaT cells stimulated with IL-22 revealed increases in NLRP3, the mature form of caspase-1 and the active form of IL-1β. HaCaT cells were treated with IL-22 (20 ng ml/1) for 24 h at 37°C. The cells were lysed and the whole-cell extracts were detected with anti-NLRP3, anti-caspase-1 and anti-IL-1β antibodies. Representative blots of three independent experiments are shown. Results were normalized against β-actin expression.

To explore the functional role of IL-22 in the production of proinflammatory mediators, some of IL-1β-induced inflammatory mediators were determined, including CXCL1, CXCL5, CXCL8, BD1, LL37, S100A7, S100A8, S100A9 and proteins of the matrix metalloproteinase 3 (MMP3). IL-22 stimulation increased transcription of these mediators (Figure 1A). According to the literature, the IL-1β facilitates specific inflammatory mechanism of IL-22 in the skin disorders. As shown in Figure 1B, IL-22 increased expression level of NLRP3, caspase-1, and mature IL-1β. This revealed that IL-22 might provoke NLRP3-mediated pathway to increase IL-1β secretion.

### IL-22–IL-22R system participates in the process of IL-1β production via the NLRP3 pathway

**Figure 2:**
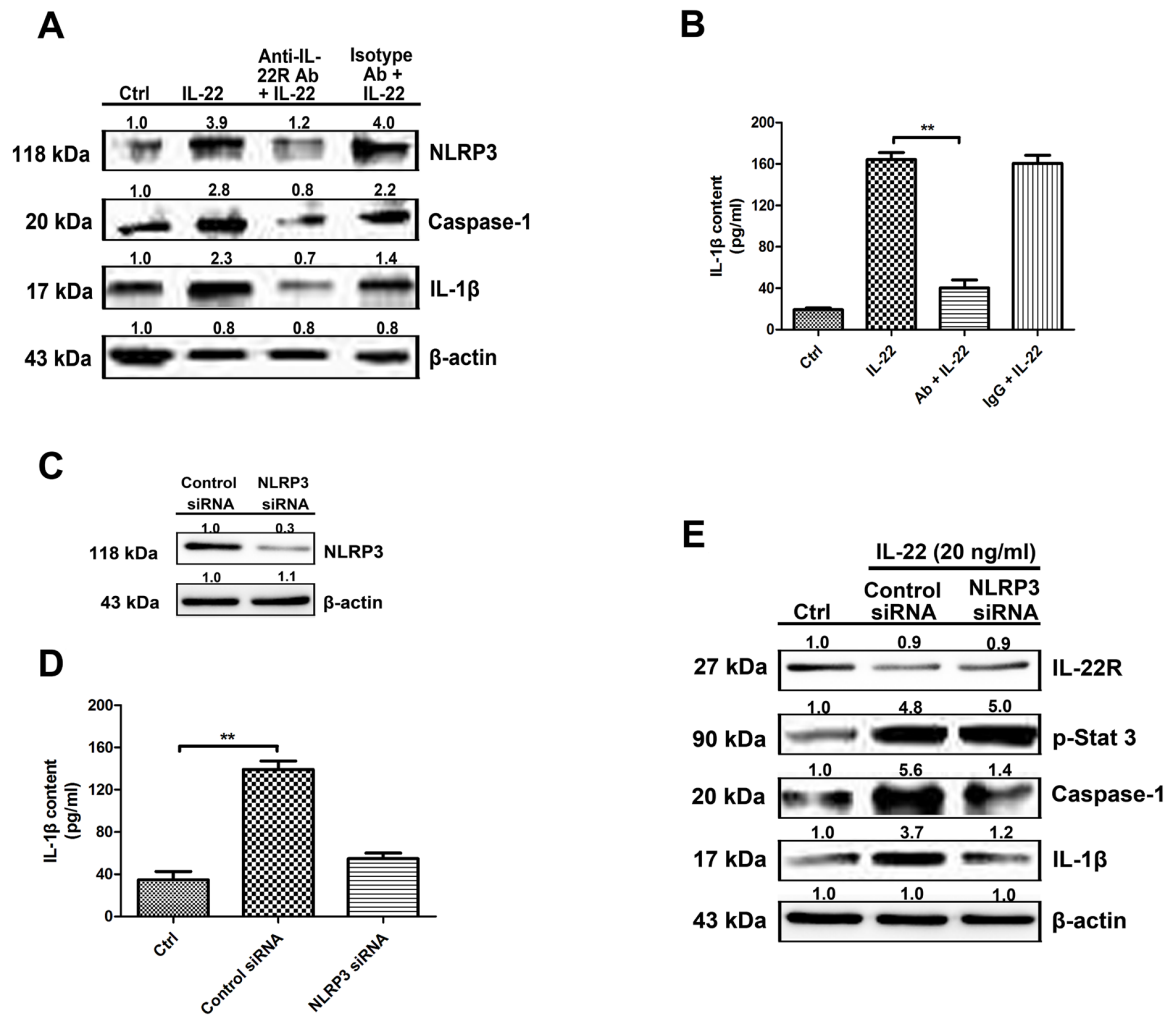
IL-22–IL-22R system participates in the process of IL-1β production via the NLRP3 pathway **(A)** To block IL-22R-mediated signaling, pre-treatment of HaCaT cells with 50 μg/ml of anti-IL-22R blocking antibody for 2 h prior to IL-22 stimulation resulted in reduced NLRP3 and caspase-1 expression **(B)** as well as a consequent reduction in IL-1β secretion as measured by ELISA. **(C)** To reduce NLRP3 expression in HaCaT cells, we transfected NLRP3 siRNA or control siRNA into HaCaT cells. The level of protein was quantified to evaluate the knockdown efficiency of the endogenous NLRP3 protein in HaCaT cells. Data from three independent experiments for protein expression are shown. **(D)** The active form of IL-1β in culture supernatants was quantitated by ELISA. **(E)** NLRP3 knockdown in HaCaT cells resulted in a reduction in the levels of caspase-1 and the active form of IL-1β in IL-22-treated cells. Representative blots of three independent experiments are shown. Results were normalized against β-actin expression. The data are expressed as means ± SD (i.e. n=3) (^**^P < 0.05). Ab, anti-IL-22R blocking antibody; IgG, isotype antibody for the negative control of IL-22R antibody.

The anti-IL-22R antibody was applied to block downstream signaling. The expression of NLRP3 and caspase-1 (Figure 2A) were decreased, as well as the IL-1β production following IL-22R blockage (Figure 2B). NLRP3 knockdown experiments were performed to confirm IL-22 induced IL-1β increase via the NLRP3 pathway (Figure 2C). Caspase-1 and IL-1β expression levels stimulated with IL-22 were reduced by NLRP3 siRNA transfection, as well as IL-1β secretion in culture supernatants examined by Elisa (Figure 2D). The downstream signaling of IL-22–IL-22R system was also detected, the representative molecule, phosphorylated STAT3 expression level was not influenced obviously by NLRP3 siRNA-transfection (Figure 2E). Therefore, our data showed that the activation of caspase-1 and accompanying IL-1β production was mediated by NLRP3 participation.

### IL-22 induces IL-1β secretion by activating NF-κB to generate ROS

**Figure 3:**
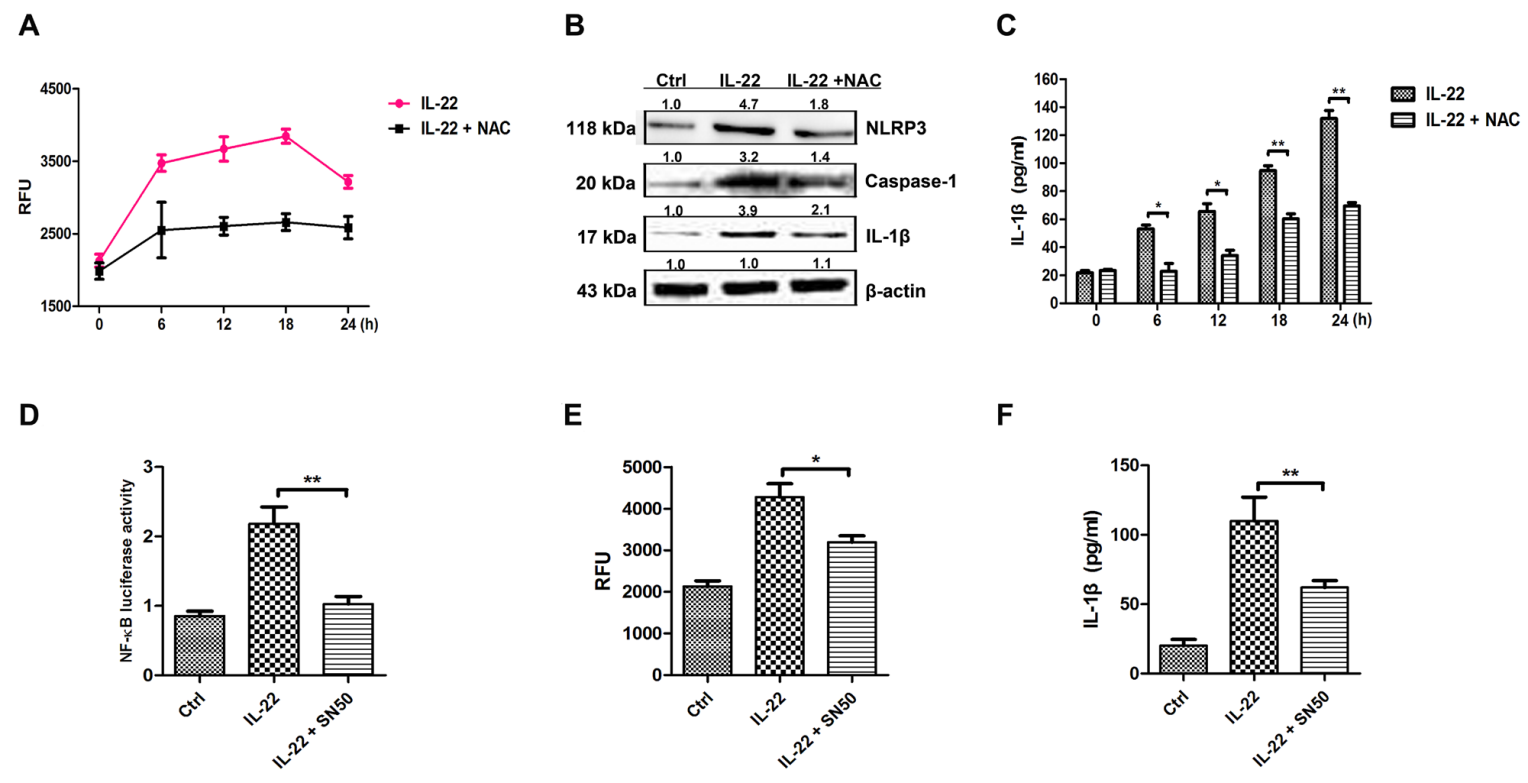
IL-22 induces IL-1β secretion by activating NF-κB to generate ROS **(A)** To measure ROS in HaCaT cells, the cells were pre-incubated for 24 h with IL-22 followed by an additional 6 h of culture with or without 5 mM of NAC. The cells were collected and ROS production was measured (represented as relative fluorescence units, RFU). **(B)** The cells were pre-incubated with IL-22 for 24 h followed by an additional 6 h of culture with or without 5 mM of antioxidant NAC. The cell lysates were collected and the expression levels of NLRP3, the mature form of caspase-1 and the active form of IL-1β were measured via immunoblotting. **(C)** The active form of IL-1β was quantitated via ELISA in the presence or absence of NAC treatment. **(D)** Treatment with the SN50 peptide abolished the activation of NF-κB following IL-22 treatment as measured by a luciferase reporter assay. **(E)** Upon SN50 treatment, ROS production (represented as RFU) as well as the levels of active IL-1β stimulated by IL-22 were reduced in HaCaT cells. **(F)** The active form of IL-1β was quantitated via ELISA in the presence or absence of SN50 treatment. Results shown are means ± SD (i.e. n=3). Data were analyzed by One-Way Analysis of Variance (ANOVA) followed by post hoc Tukey test. ^*^P < 0.05, ^**^P < 0.01, compared with control.

It has been reported that ROS can activate the NLRP3 inflammasome [29]. The ROS level was measured after IL-22 treatment in this study. As results showed that IL-22 promoted ROS formation significantly (Figure 3A). The role of ROS in activating NLRP3-caspase-1 pathway to produce active IL-1β was determined by the application of NAC (Figure 3A). NAC blocked the generation of ROS induced by IL-22, accompanying the decrease of NLRP3, caspase-1 and IL-1β expressions (Figure 3B and 3C). Additionally, IL-22 induces NF-κB activation (Figure 3D). Pretreated with the NF-κB inhibitor SN50 resulted in the reduction of NF-κB levels (Figure 3D) and the generation of ROS (Figure 3E). These results demonstrate that IL-22 induces ROS production via NF-κB activation.

### IL-22 inhibits PAR-2 expression in HaCaT keratinocytes

**Figure 4:**
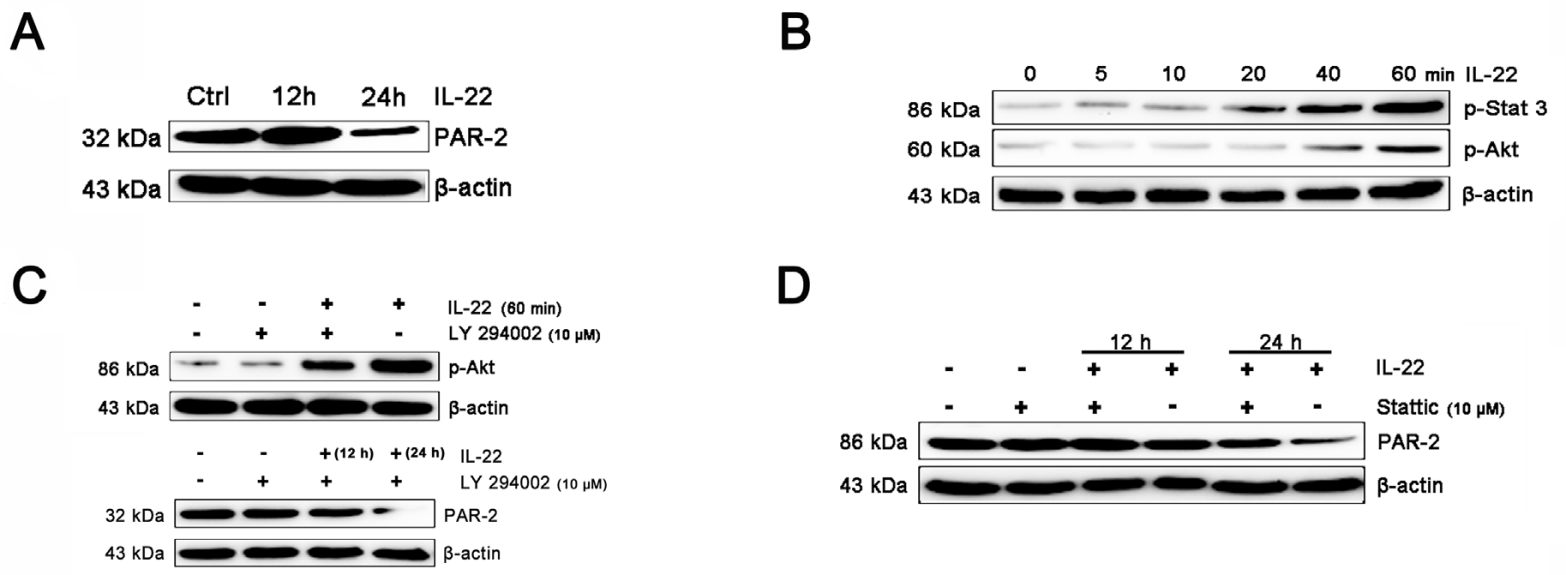
IL-22 inhibits PAR-2 expression in HaCaT keratinocytes **(A)** Addition of IL-22 (20 ng/mL) to keratinocytes for 24 h inhibited PAR-2 expression. **(B)** Addition of IL-22 (20 ng/mL) to keratinocytes induced phosphorylation of Stat3, Akt. Western blot of melanocytes lysates after 0, 5, 10, 20, 40, 60 min of IL-22 treatment (20 ng/mL). **(C)** Cultured cells were pre-treated with 10 μM of LY294002 for 60 min, followed by exposure to IL-22 (20 ng/mL) for 60 min. The effect of IL-22 on phosphorylation of Akt was partially suppressed by LY294002. Cultured cells were pre-treated with 10 μM of LY294002 for 60 min, followed by exposure to IL-22 (20 ng/mL) for 12 or 24 h, the expression of PAR-2 were examined. **(D)** Cultured cells were pre-treated with 10 μM of Stattic for 60 min, followed by exposure to IL-22 (20 ng/mL) for 12 h and 24 h. The inhibition of IL-22 on PAR-2 expression can be interfered by Stattic. Results were normalized against β-actin expression.

PAR-2 is expressed on keratinocyte, which can regulate melanosome transfer [30]. We examined the effect of IL-22 on PAR-2 expression of keratinocytes. As shown in Figure 4A, treatment of IL-22 for 24 h decreased PAR-2 expression. We studied the response of keratinocytes to treatment with IL-22 (20 ng/mL) over time ranging from 5 min to 60 min and observed rapid phosphorylation of Stat-3 beginning at 20 min after IL-22 treatment (Figure 4B). These results demonstrated that melanocytes responded directly to IL-22 stimulation by activating the JAK/Stat-3 signaling pathway. IL-22 also engaged the PI3K/Akt pathway, as the phosphorylation of Akt with peak activation at 60 min following IL-22 treatment (Figure 4B). The effect of IL-22 on Akt was partially suppressed by pre-treatment of keratinocytes with LY294002 (Figure 4C). The inhibition of IL-22 on PAR-2 expression can be interfered by Stat-3 inhibitor Stattic, but not LY294002 (Figure 4C and 4D). From these results, we drew a conclusion that the activation of Stat-3 was essential for the effect of IL-22 on PAR-2 expression, but not the invoking of Akt.

### Effect of IL-22 on melanogenesis of NHEM

**Figure 5:**
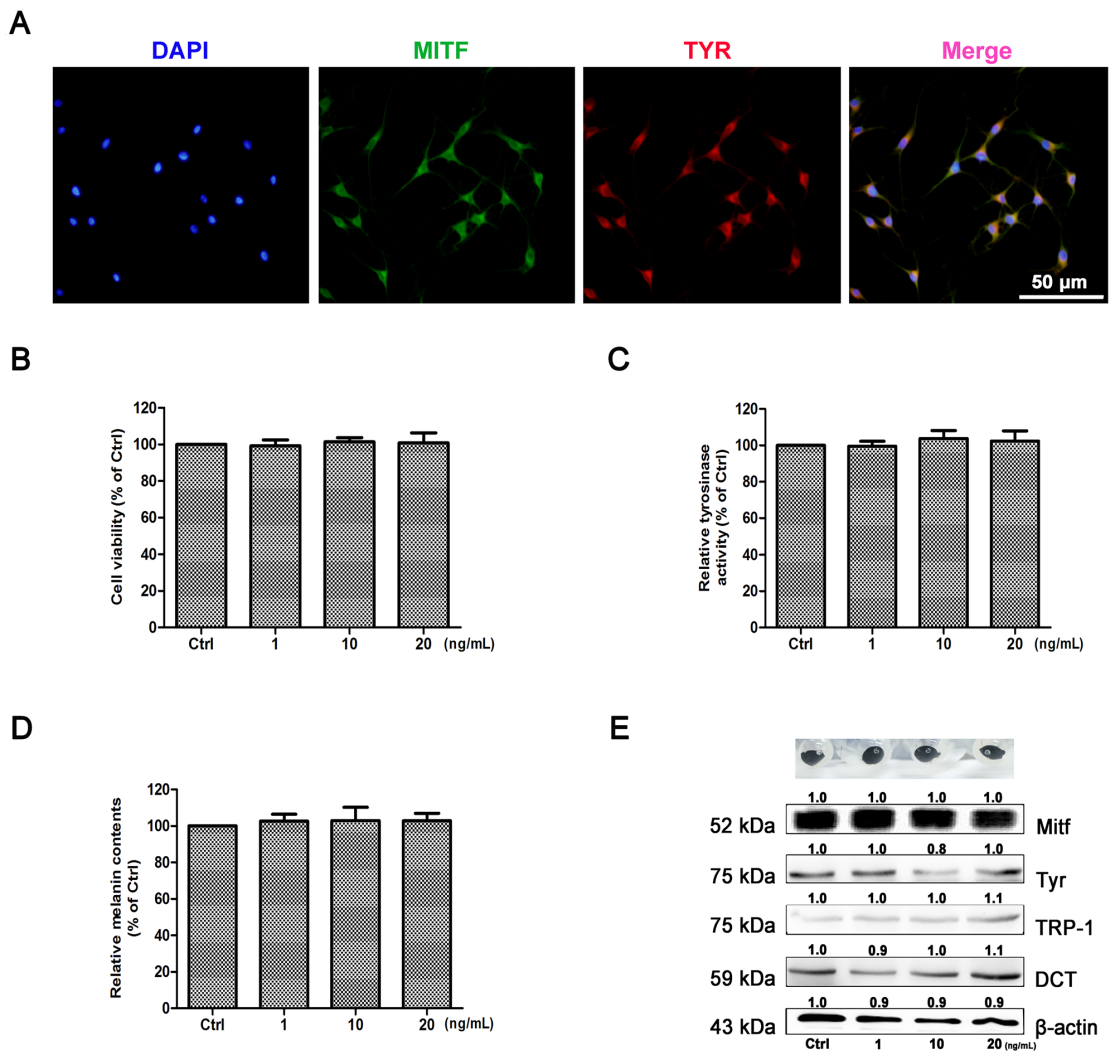
Effect of IL-22 on melanogenesis of NHEM **(A)** Identification of primary melanocytes. Cells were labeling with Mitf (green) and Tyr (red) antibodies. Nuclei were labeled with DAPI (blue). **(B)** After incubation with IL-22 (0, 1, 10, 20 ng/ml) for 48 h, cell viability was determined using a MTT assay. **(C)** Tyrosinase activity was determined by _L_-DOPA oxidation as described in ‘Materials and methods’. Stimulation of tyrosinase activity of NHEM with IL-22 (0, 10, 20 ng/ml) for 48 h. **(D)** Melanin content was performed as described in ‘Materials and methods’ the same for 48 h. **(E)** Western blot assays were performed to examine Mitf, Tyr, TRP-1 and DCT expression levels, Total cellular proteins (20 μg/lane of NHEM) were subjected to 10% SDS-PAGE. Results were normalized against β-actin expression. Results shown are means ± SD (i.e. n=4). Data were analyzed by One-Way Analysis of Variance (ANOVA) followed by post hoc Tukey test.

As shown in Figure 5A, the purity of cells was identified by immunohistochemical staining with two markers of Tyr and Mitf, and 99% of primary melanocytes were obtained. To determine the effect of IL-22 on melanogenesis, MTT assay was performed as the first step to examine whether IL-22 was cytotoxic to NHEM. As shown in Figure 5B, IL-22 was not cytotoxic to NHEM within certain limits. NHEM was then exposed to different concentrations of IL-22, tyrosinase activity was measured by the rate of _L_-DOPA oxidation. As comparing with control group, IL-22 made no effect on the activity of Tyr (Figure 5C), as well as the melanin synthesizing level in NHEM (Figure 5D). The pigment cell-specific melanosomal proteins Mitf, Tyr, TRP-1 and DCT were not responding to IL-22 stimulation (Figure 5E).

### Effect of conditional medium on melanogenesis of NHEM

**Figure 6:**
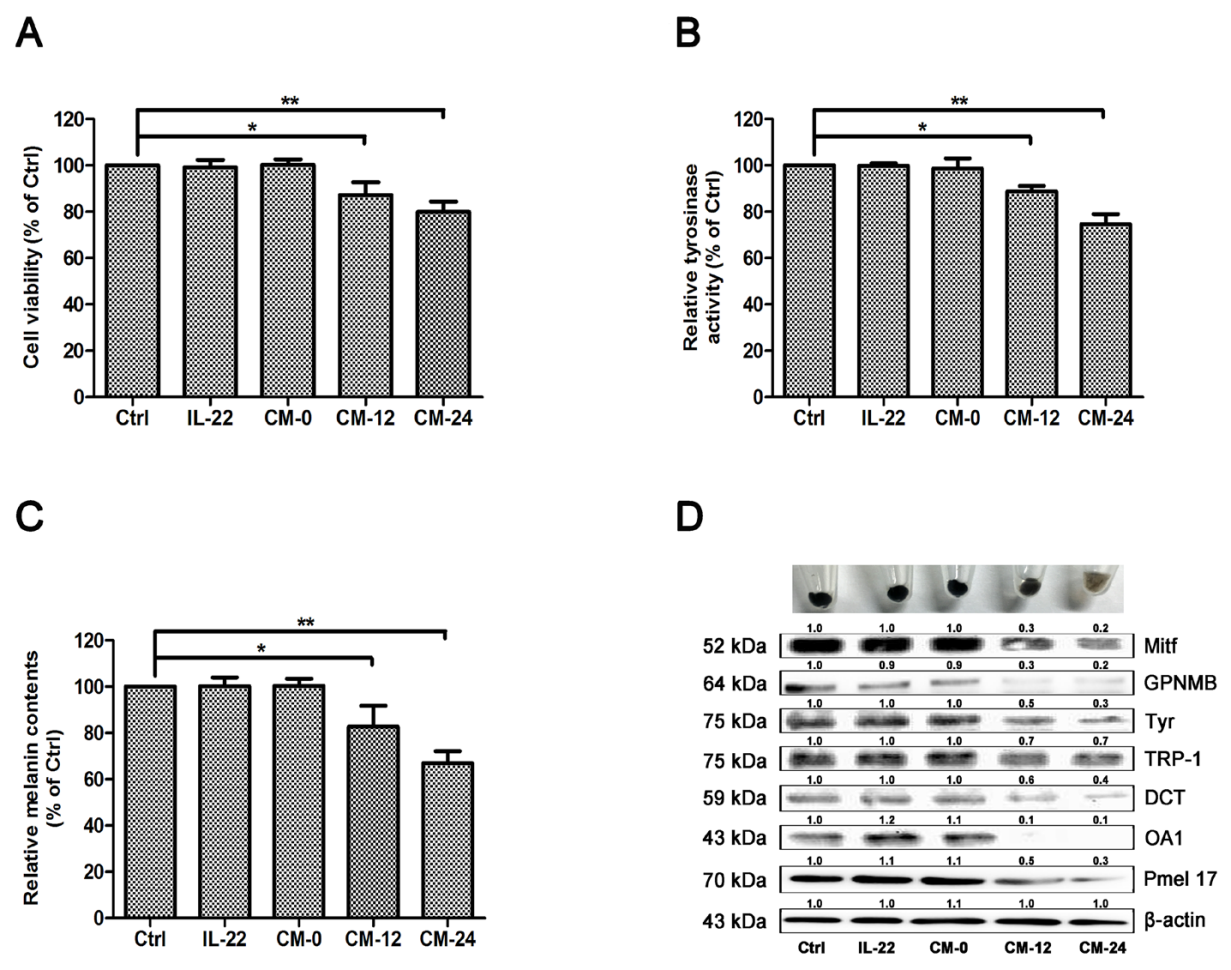
Effect of conditional medium on melanogenesis of NHEM **(A)** After incubation with IL-22 and different conditional medium for 48 h, cell viability was determined using a MTT assay. **(B)** Tyrosinase activity was determined by _L_-DOPA oxidation as described in ‘Materials and methods’. **(C)** Melanin content was performed as described in ‘Materials and methods’. **(D)** Western blot assays were performed to examine GPNMB, Mitf, Tyr, TRP-1, DCT, OA1 and Pmel17 expression levels, Total cellular proteins (20 μg/lane of NHEM) were subjected to 10% SDS-PAGE. Results were normalized against β-actin expression. Results shown are means ± SD (i.e. n=3). Data were analyzed by One-Way Analysis of Variance (ANOVA) followed by post hoc Tukey test. ^*^P < 0.05, ^**^P < 0.01, compared with control.

Among skin cell populations, keratinocytes are the unique major targets of IL-22. In consideration of the interactive relationship between keratinocytes and melanocytes, we examined the effect of IL-22–treated keratinocytes conditional medium on melanocytes. Three control groups were set, including medium treated only group (Ctrl), 20 ng/ml of IL-22 treated group and conditional medium with no IL-22 treated group (CM-0). Two administered groups were 12 h–IL-22–treated conditional medium group (CM-12) and 24 h–IL-22–treated conditional medium group (CM-24). After 48 h of treatments, MTT assay was performed as the first step. CM-12 and CM-24 showed certain extent suppression on melanocytes (Figure 6A). Then TYR activity and melanin content were measured. CM-12 and CM-24 inhibited the activity of Tyr (Figure 6B) and restrained melanogenesis (Figure 6C). Furthermore, CM-12 and CM-24 suppressed expressions of the melanin synthesis related proteins (Figure 6D).

### Conditional medium inhibits NHEM migration

**Figure 7:**
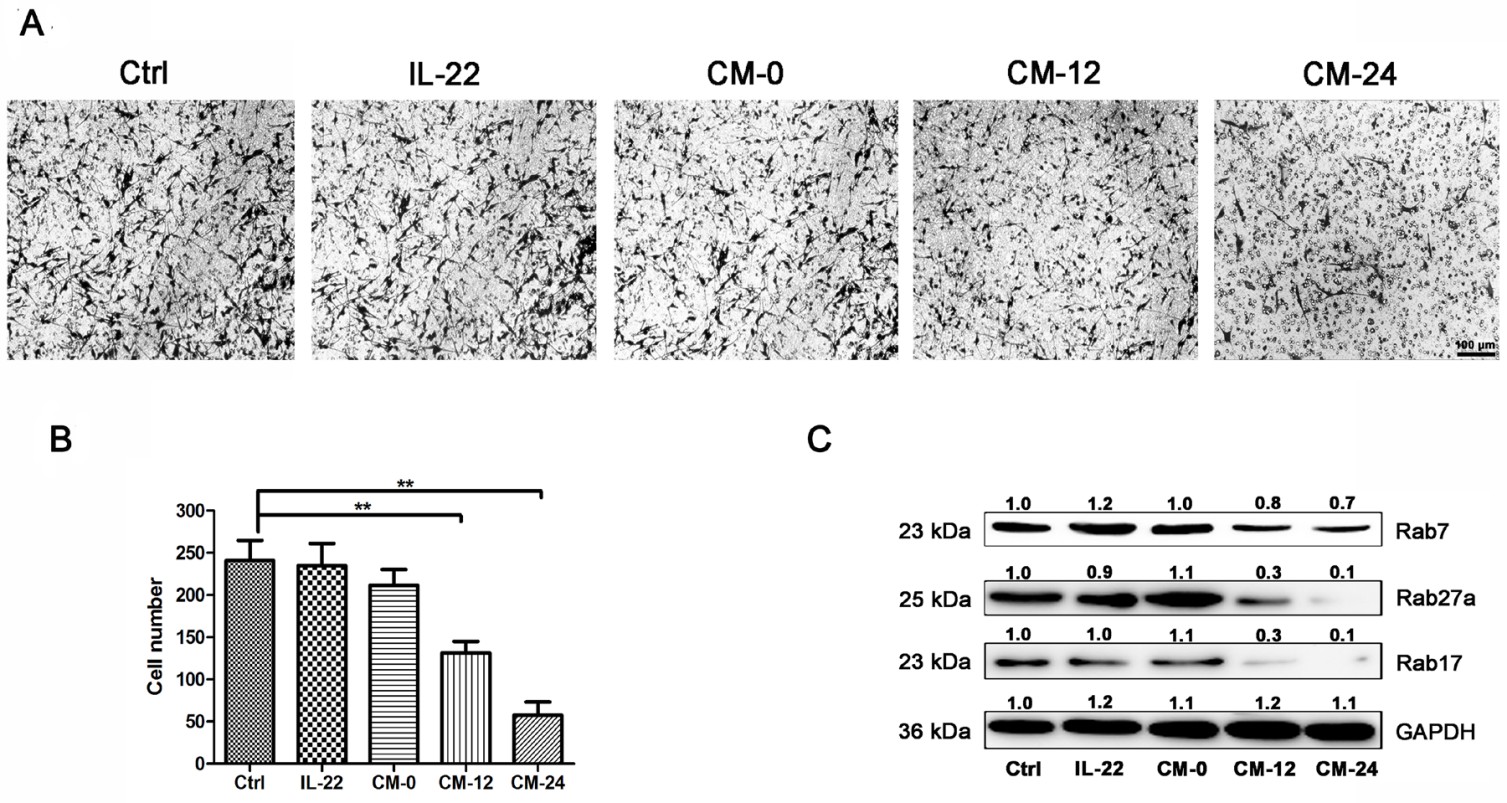
Conditional medium inhibits NHEM migration **(A)** NHEM cells were seeded in the upper chamber of transwell. CM-12 and CM-24 inhibited NHEM migration. Scale bar, 50 μm. **(B)** After 24 h, the migrated NHEMs were quantified. **(C)** Western blot assays were performed to examine Rab7, Rab27a and Rab17 expression levels, Total cellular proteins (20 μg/lane of NHEM) were subjected to 12 % SDS-PAGE. Results were normalized against GAPDH expression. Results shown are means ± SD (i.e. n=3). Data were analyzed by One-Way Analysis of Variance (ANOVA) followed by post hoc Tukey test. ^**^P < 0.01, compared with control.

The effects of conditional medium on NHEM migration were observed. As shown in Figure 7A,B, CM-12 and CM-24 inhibited the cells migration. It has been reported that Rab GTPases play roles in melanosome maturation or trafficking in melanocytes. Researches have been published showing that Rab7 mainly regulates early and intermediate stage melanosomes and Rab27a primarily work on intermediate and mature melanosomes [31]. In addition, Rab17 acts on melanosomes downstream of Rab27a [32]. Our data shown that CM-12 and CM-24 suppressed expressions of the Rab7, Rab 27a and Rab17 (Figure 7C).

### Conditional medium induces NHEM apoptosis

**Figure 8:**
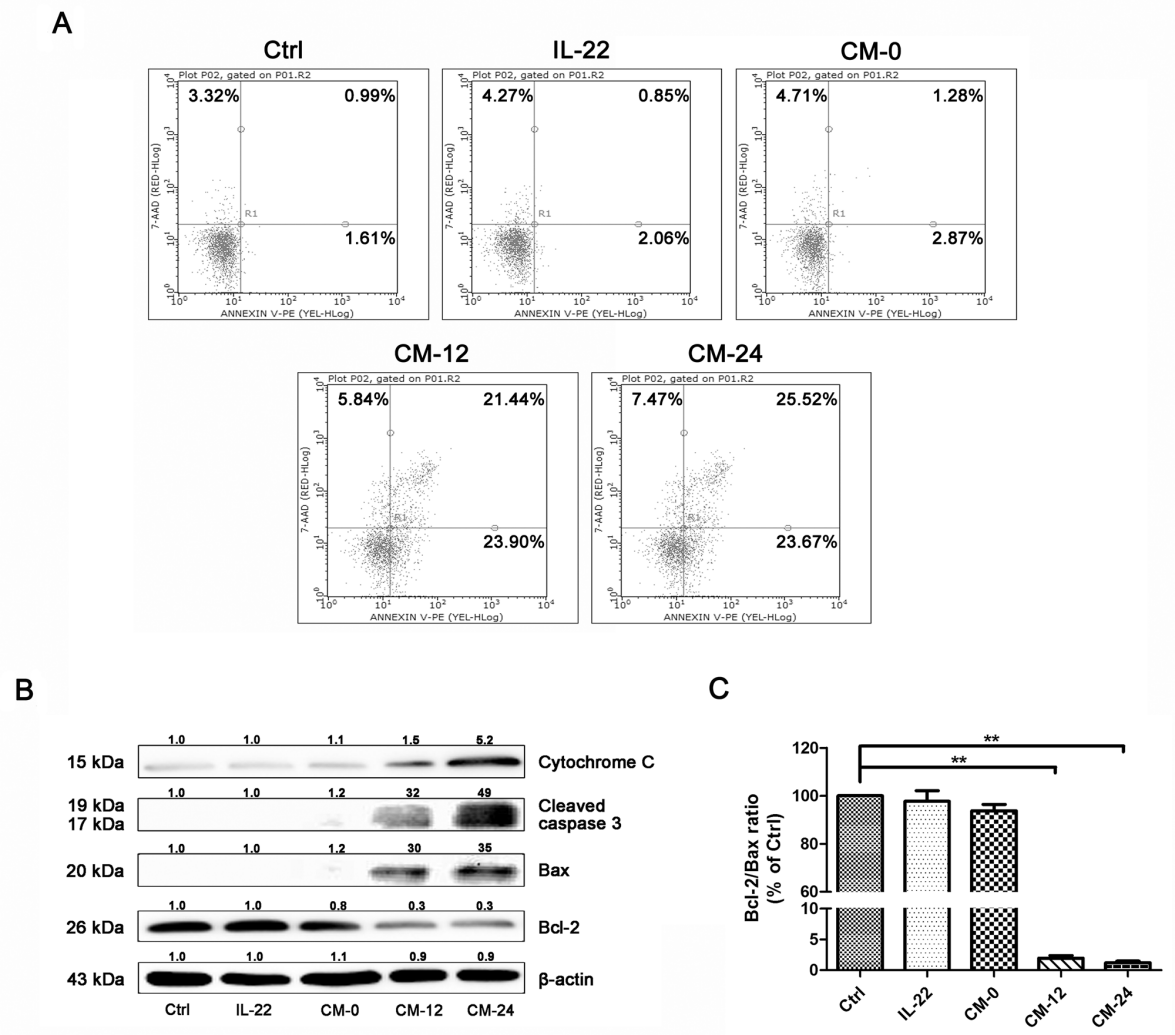
Conditional medium induces NHEM apoptosis **(A)** Apoptosis of different groups of NHME were measured using flow cytometry with double staining of Annexin V and PI. **(B)** Western blot of cytosol fraction of melanocytes demonstrated increase of Bax, cleaved-caspase 3 and cytochrome c protein levels and descrease of Bcl-2. Results were normalized against β-actin expression. **(C)** The band densities of interest proteins were measured by Tanon 5200 Multi analysis software program, and then the Bcl-2/Bax ratio were got. Results shown are means ± SD and are representative of 3 independent experiments. Data were analyzed by one-way analysis of variance (ANOVA) followed by post hoc Tukey test. ^**^P < 0.01, compared with control.

The conditional medium induced NHEM apoptosis, as shown in Figure 8A, the percentage of apoptotic cells increased significantly in CM-12 and CM-24 group by flow cytometry analysis. In our studies, CM-12 and CM-24 increased the expresssions of Bax, cleaved-caspase 3 and cytochrome c, at the same time decreased Bcl-2 (Figure 8B), hence, Bcl-2/Bax ratio significantly decreased (Figure 8C).

## DISCUSSION

In mammals, the epidermis forms the top protective covering of skin to provide photoprotection and thermoregulation. This function relies on the principal trait of epidermal melanocytes——‘pigmentation’. Melanin, the pigment responsible for most pigmentation, is most contained by epithelial cells [33]. Except those of the eye producing their own melanin, most pigmented epithelial cells acquire their pigment from melanocytes, such as keratinocytes. On the basis of physiological structure, melanocytes are located in the basal layer of the epidermis, surrounding by approximately 36 keratinocytes. Melanocytes and keratinocytes establish a division of activities, that one cell producing pigment, another cell using it [34, 35].

There were literatures reported that Rab7 is involved in the transport of late endosomes/lysosomes [36, 37]. The activated Rab27a can be found on mature melanosomes, which locate in the tips of the dendrites of melanocytes [38–40]. Rab17 resides on melanosome and endosomes (REs) membranes in melanocytic cells, which also regulates melanosome trafficking. It has been proved that Rab27a works as upper stream of Rab 17 on melanosome transport [32]. Multiple enzymatic and structural proteins participate in regulating the maturation of melanosomes, including Tyr, TRP-1, DCT, OA1, antigen recognized by T cells (MART-1), Pmel17/gp100, GPNMB and others [41–43]. The transcription of GPNMB is regulated by MITF [44, 45], which also regulates other pigmentation genes, such as Tyr, TRP-1, DCT, Pmel17, OA1 and so on.

Vitiligo is an acquired depigmentary skin disorder, which is not only a disease of skin melanocytes. Recent research in vitiligo suggests that skin immune innate system participates in the pathogenetic process and many triggers precede adaptive immune responses targeting melanocytes [46]. Furthermore, various cytokines released from keratinocytes chronic involve in the immune responses of the epidermal layer. It has been reported that the mRNA levels of IL-1β in vitiligo patients were found to be significantly higher than controls suggesting its possible role in inflammatory pathogenesis of vitiligo [47]. It has been reported that in comparison with controls, tissue level of IL-22 in lesional skin were significantly higher, being further augmented in perilesional skin. IL-22 increased IL-1β level via ROS production which leaded to NLRP3 activation in HaCaT cells (Figure 3). These results indicate that IL-22 regulates IL-1β production via NLRP3-caspase-1 activation (Figure 2). IL-22 also increased the production of anti-microbial peptides and chemokines from HaCaT cells (Figure 1). We demonstrated that IL-22 played little parts in cell proliferation and melanogenesis of melanocytes (Figure 5), and were identical with those reported in literature that melanocytes showed no coexpression of IL-22RA1 and IL-10R2 [48]. We treated HaCa T keratinocytes with IL-22 (20 ng/ml) for 12h and 24h, and then collected the conditional medium (CM-0, CM-12 and CM-24). CM-12 and CM-24 suppressed expressions of the melanin synthesis related proteins at different levels, including Mitf, GPNMB, Tyr, TRP-1, DCT, OA1, and Pmel 17 (Figure 6). Considering the involvement of the keratinocyte receptor PAR-2 in melanosome transfer, we examined the effect of IL-22 on PAR-2 expression of keratinocytes. As shown in Figure 4A, treatment of IL-22 for 24 h decreased PAR-2 expression. We studied the response of keratinocytes to treatment with IL-22 (20 ng/mL) over time ranging from 5 min to 60 min and observed rapid phosphorylation of Stat-3 beginning at 20 min after IL-22 treatment. These results demonstrated that kerationocytes responded directly to IL-22 stimulation by activating the JAK/Stat-3 signaling pathway. IL-22 also engaged the PI3K/Akt pathway, as the phosphorylation of Akt with peak activation at 60 min following IL-22 treatment (Figure 4B). The effect of IL-22 on Akt was partially suppressed by pre-treatment of keratinocytes with the PI3K inhibitor, LY294002 (Figure 4C). The inhibition of IL-22 on PAR-2 expression can be interfered by Stat-3 inhibitor Stattic, but not LY290042 (Figure 4C and 4D). We speculated that IL-22 modulated PAR-2 activity to decrease melanosome transfer and affected pigmentation. This effect further illustrated the suppression on melanosomes mature and trafficking. As shown in Figure 7, CM-12 and CM-24 inhibited the cells migration. The effect of conditional medium on NHEM apoptosis was the next focus of our study. As shown in Figure 8A, the percentage of apoptotic cells increased significantly in CM-12 and CM-24 group by flow cytometry analysis. CM-12 and CM-24 induced the expresssions of Bax, cleaved-caspase 3 and cytochrome c, at the same time reduced Bcl-2 (Figure 8B), hence, the Bcl-2/Bax ratio was significantly decreased (Figure 8C).

**Figure 9:**
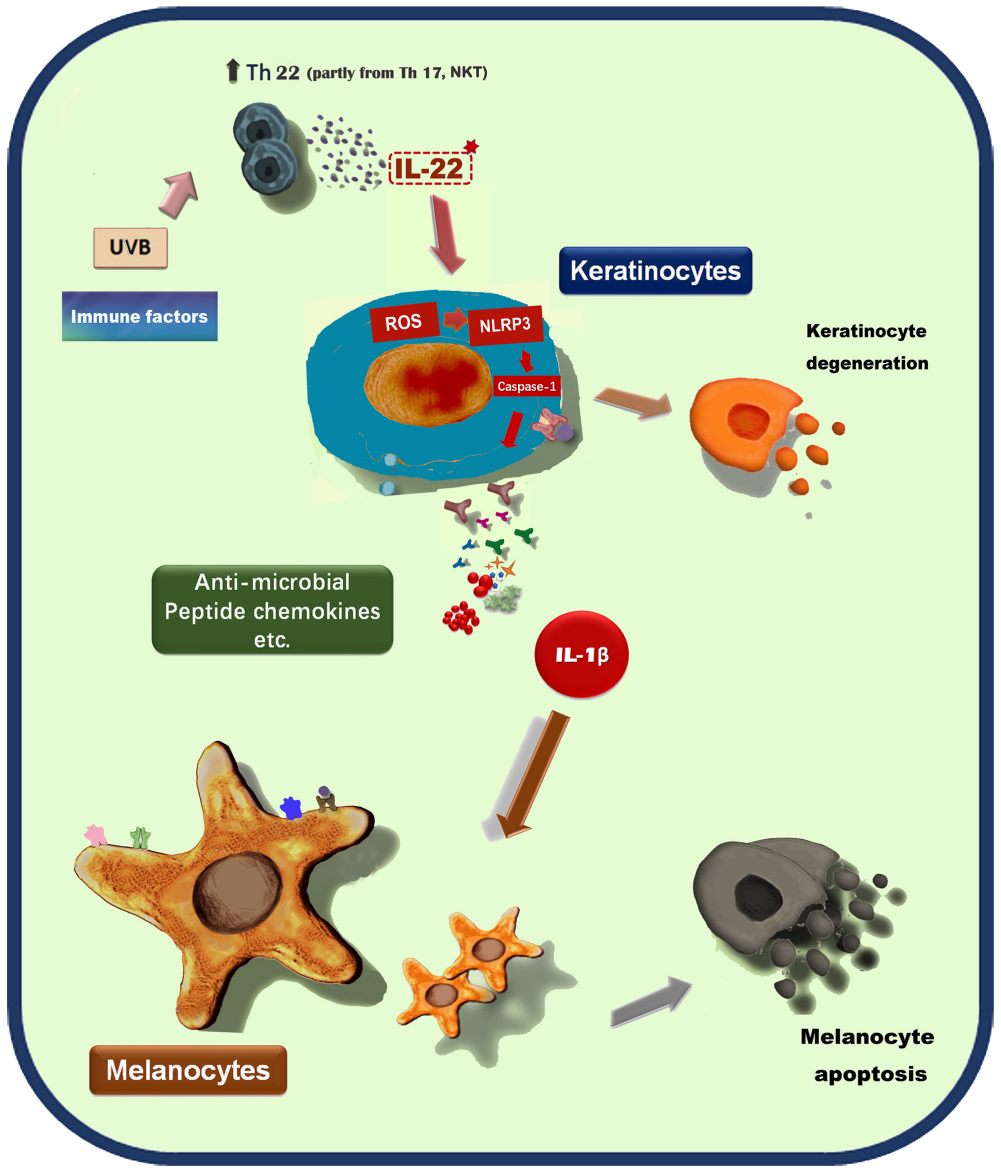
A proposed scheme shows the indirect effect of IL-22 on melanocytes by keratinocytes participation

In conclusion, our data demonstrated that IL-22 could indirectly regulate melanocytes functions by keratinocytes participation and mediation (Figure 9). The components released by keratinocytes contained in the conditional medium and their action mode, maybe individual or synergistic, need intense researches. This is another evidence of cell-cell connections to regulate the body’s function.

## MATERIALS AND METHODS

### Reagents

Recombinant human interleukin-22 was from R&D (USA). Dimethylsulfoxide (DMSO), L-3,4-dihydroxyphenylalanine (_L_-DOPA), melanin, LY294002, β-actin primary antibody and horseradish peroxidase-conjugated secondary antibodies were purchased from Sigma-Aldrich (USA). Stattic was from TOCRIS (USA). Medium 254 and Human Melanocyte Growth Supplement (HMGS) were from Invitrogen (USA). GAPDH primary antibody was from Proteintech (USA). Enhanced BCA protein assay Kit, phenylmethylsulfonyl fluoride (PMSF), cell lysis buffer for Western and IP, nuclear and cytoplasmic protein extraction Kit, LDH cytotoxicity assay Kit were from Beyotime Institute of Biotechnology (China).

### Cell culture

The studies on human material were approved by local ethic committee. Normal human foreskin-derived epidermal melanocytes (NHEM) were derived from young male adult foreskins (ethnic Han/aged 18 to 22 years) obtained at circumcision following standard protocols [49]. Briefly, foreskins were cut into strips and digested with 0.25% trypsin at 4°C for 20 h. Epidermis was separated from dermis. The NHEM suspension was filtered and cells were washed twice at 1,500 rpm for 5 min prior to resuspension in Medium 254 (containing the HMGS). NHEM were grown in a humidified atmosphere with 5% CO_2_ at 37°C.

HaCaT human keratinocytes cell was obtained from CAS (Chinese Academy of Sciences, China). The cells were grown in Medium 245 supplemented with 10% fetal bovine serum (GIBCO, USA), 100 U/ml penicillin and 100 mg/ml streptomycin (GIBCO, USA) in a humidified atmosphere with 5% CO_2_ at 37°C.

### Conditional medium collection

HaCaT cells were grown in Medium 245 supplemented with 10% fetal bovine serum in 60 mm dish. After growing adhering to the wall, HaCaT cells were cultured sequentially to about 80% confluent state. Then cells were rinsed twice with PBS and divided into three groups. Cells of group one were cultured in vehicle treatment for 24 hours; cells of group two were firstly cultured in serum-free Medium 245 for 12 hours and then IL-22 (20 ng/ml) was added, for another 12 hour; cells of group three were cultured in 3 ml of serum-free Medium 245 containing IL-22 (20 ng/ml) for 24 h. The different time-point cultural treated supernatant was collected and centrifuged at 1,500 rpm for 5 min. Aspirating 2 ml of the liquid supernatant and storing in 4°C (we used it immediately after collection, otherwise, it need to be frozen and stored at −70°C until use). According to the different treated time-point, the conditional medium was termed CM-0 (from group one), CM-12 (from group two) and CM-24 (from group three).

### Immunofluorescence

NHMC were grown on coverslips that had previously placed in 24-well plates. After the cells grew adhering to the wall, they were fixed in 4% paraformaldehyde PBS for 30 min at RT and permeabilized with 0.03% Triton X-100 in PBS for 5 min. The cells were rinsed with 0.1% TBST, blocked for 30 min, and incubated for 2 h at room temperature with anti-Tyr and anti-Mitf primary antibodies. Cells were rinsed three times with TBST and incubated for 60 min at room temperature with FITC-conjugated and TRITC-conjugated secondary antibodies. After further rinsing, the coverslips were sealed and the nuclei were stained with DAPI. Images were captured using a CCD camera (Olympus CKX41, Tokyo, Japan).

### Tyrosinase activity and melanin contents assay

Tyrosinase activity, as the dopa oxidase here, was measured by the rate of _L_-DOPA oxidation as reported [50]. NHEM were treated with IL-10 for 48 h, washed with ice-cold PBS, lysed by incubation in cell lysis buffer [1 mM PMSF] at 4°C for 20 min, and then lysates were centrifuged at 14,000 rpm for 10 min to obtain the supernatant for activity assay and centrifugation for melanin contents assay. Protein concentrations were determined by BCA kit with bovine serum albumin (BSA) as a standard. 100 μL of supernatant containing the same 10 μg total proteins was added to each well in 96-well plate, and then mixed with 100 μL 0.1% L-DOPA in 0.1 M PBS (pH 6.8) (M/V). After incubation at 37°C for 0.5 h, the dopachrome was monitored by measuring the absorbance at 475 nm.

Total melanin in the cell pellet was dissolved in 100 μL of 1 N NaOH/10% DMSO for 1 h at 80°C, and solubilized melanin was measured at 405 nm. Melanin content was calculated as a percent of the control.

### Real time–PCR

The primers employed in real-time PCR were as follows: 5’-TGAGATGGCCTCAGGTGGTA-3’ and 5’-CG GGCAGGCAGAATAGAGAC-3’ for human b-defensin (BD1); 5’- GGGGCTCCTTTGACATCAGT-3’ and 5’-TG GGTACAAGATTCCGCAAA-3’ for human LL37; 5’-GCA TGATCGACATGTTTCACAAATACAC-3’, and reverse, 5’- TGGTAGTCTGTGGCTATGTCTCCC-3’ for human S100 A7; 5’-GCCGTCTACAGGGATGACCT-3’ and 5’-TTTGT GGCTTTCTTCATGGC-3’ for human S100A8; 5’-CAAA GAGCTGGTGCGAAAAG-3’ and 5’-CGAAGCTCAGCTG CTTGTCT-3’ for human S100A9; 5’-GCTGCTCCTGCTC CTGGTA-3’ and 5’-CTTTCCGCCCATTCTTGAGT-3’ for human CXCL1; 5’-TGCGTTGCGTTTGTTTACAG-3’ and 5’-GAAAAGGGGCTTCTGGATCA-3’ for human CXCL5; 5’-GCAGCTCTGTGTGAAGGTGC-3’ and 5’-TCTGCACC CAGTTTTCCTTG-3’ for human CXCL8; 5’-TGGCATTC AGTCCCTCTATGG-3’, 5’-AGGACAAAGCAGGATCA CAGTT-3’ for human matrix metalloproteinase 3 (MMP-3).

### Western blot analysis

The protein suspension was obtained as the method mentioned above. Western blot was performed as described previously [51]. The primary antibodies used were p-Stat-3 (CST 9145), MITF (BS1550), TYR (C-19) (SC7833), TRP-1 (SC10443), DCT (ab74073), GPNMB (ab125898), OA1 (ab209537), Pmel 17 (ab52058), PAR-2 (CST 6976), Rab7 (ab137029), Rab27a (ab55667), Rab17 (ab118998), NLRP3 (ab16097), caspase-1 (CST 2225), IL-1β (CST 2022), IL-22R (ab5984), cytochrome c (CST 11940), Bcl-2 (CST2870), Bcl-x_L_ (CST 2764), Bax (CST 2772), cleaved-caspase 3 (CST 9661), GAPDH (60004) and β-actin (CST3700). Proteins were visualized using an enhanced chemiluminescence detection system. Densitometric analysis was again performed by using the Tanon 5200 Multi to scan the signals. Western blot assay results reported here are representative of at least 3 experiments.

### Reactive oxygen species measurement

ROS generation was detected by 2’,7’-dichlorodihydrofluorescein (D6883, Sigma), which can be oxidized to fluorescent dichlorodihydrofluorescein due to ROS. N-acetyl-L-cysteine (NAC, 3 mM, A9165, Sigma) was applied for 4 h after stimulation with IL-22 to inhibit ROS generation. In a separate experiment, HaCaT cells were treated with 20 ng/ml of IL-22 in a time dependent manner, for 6, 12, 18 and 24 h. Cells were incubated with 10 μM 2’,7’-dichlorodihydrofluorescein for 30 min, then the cell lysis buffer was added. After 5 min, 150 μl of the mixture was used for the fluorescence emission at 485 nm. SN50 is a nuclear factor-kappa B (NF-κB) inhibitor (10 μM, SML1471, Sigma). HaCaT cells were treated with 20 ng/ml of IL-22 for 24 h, with or without pretreatment with SN50.

### IL-1β content determination

The culture supernatants of HaCaT cells were collected and the levels of secreted IL-1β were determined using an ELISA kit in accordance with the manufacturer’s recommended protocols (557953; BD Bioscience). The detection limit of IL-1β was 4 pg/ml.

### NF-κB luciferase assay

HaCaT cells (2×10^5^) were plated in 24-well tissue culture plates and transfected with 2 μg of NF-κB-dependent luciferase reporter plasmid (Stratagene, USA). The cells were treated with IL-22 for 24 h at 37°C and then lysed in 100 μl of passive lysis buffer (Promega). 40 μl of cell lysates were assayed for both firefly and Renilla luciferase activities using the Dual-Luciferase Reporter Assay System (Promega) in accordance with the manufacturer’s instructions, and the relative light units (RLU) were measured on a luminometer. RLU from firefly luciferase was normalized for transfection efficiency to the Renilla luciferase RLU in each lysate (normalized RLU = RLU _firefly luciferase_/RLU _Renilla luciferase_).

### Blocking of IL-22 receptor signaling

The cells were pretreated with either rabbit anti-human IL-22R antibody (AF2770; R&D systems) or with the isotype control (AB-108-C; R&D systems) at a concentration of 50 μg/ml for 2 h to block IL-22R in HaCaT cells.

### siRNA silencing of NLRP3 expression

To reduce endogenous NLRP3 expression, siRNA oligonucleotides (80 pmol, sc-45469; Santa Cruz) was used according to the manufacturer’s instructions. The cells were transfected with either NLRP3 siRNA oligonucleotide or a non-targeted control siRNA oligonucleotide and incubated for 6h at 37°C under standard culture conditions. Then the cells were cultured with fresh culture media for further 14 h. In the next moment, the cells were treated with 20 ng/ml of IL-22 for another 24h. Thereafter, the cell lysates were harvested for immunoblotting, the supernatants for Elisa assay.

### Flow cytometry analysis

NHMC (2 × 10^5^/well) grown were in 6-well plate were treated according to above description. After the treatment, NHMC were harvested and analyzed for cell apoptosis by Annexin-V and propidium iodide (PI) staining, using FITC Annexin-V apoptosis detection kit (Life technology) according to the manufacturer’s instructions with a flow cytometry (BD, USA).

### Transwell migration assay

The bottom chambers of Transwell were set as five different groups (Ctrl, IL-22, CM-0, CM-12 and CM-24 group), and the top chambers were seeded inactivated 5 × 10^4^ cells per well NHEM in 200 μl Medium 254 (containing the HMGS). After 24 h of migration, the cells on the top surface of the membrane (non-migrated cells) were scraped with a cotton swab and the cells spreading on the bottom sides of the membrane (migrated cells) were fixed with cold 4% paraformaldehyde for 30 min. After that, those migrated cells were stained with 0.1% hexamethyl pararosaniline. Images were taken using Olympus inverted microscope and migrated cells were quantified by manual counting.

### Statistical analysis

All data were expressed as means ± SEM. Statistical analysis was performed with one-way ANOVA followed by Tukey’s post hoc test for multiple comparisons tests. Significant differences were accepted when *P* < 0.05.

## References

[1] Slominski A (2005). Neuroendocrine system of the skin. Dermatology.

[2] Costin GE, Hearing VJ (2007). Human skin pigmentation: melanocytes modulate skin color in response to stress. FASEB J.

[3] Skobowiat C, Dowdy JC, Sayre RM, Tuckey RC, Slominski A (2011). Cutaneous hypothalamic-pituitary-adrenal axis homolog: regulation by ultraviolet radiation. Am J Physiol Endocrinol Metab.

[4] Weiss E, Mamelak AJ, La Morgia S, Wang B, Feliciani C, Tulli A, Sauder DN (2004). The role of interleukin 10 in the pathogenesis and potential treatment of skin diseases. J Am Acad Dermatol.

[5] Richmond JM, Frisoli ML, Harris JE (2013). Innate immune mechanisms in vitiligo: danger from within. Curr Opin Immunol.

[6] Trifunović J, Miller L, Debeljak Ž, Horvat V (2015). Pathologic patterns of interleukin 10 expression – A review. Biochem Med.

[7] Attili VR, Attili SK (2008). Lichenoid inflammation in vitiligo-a clinical and histopathologic review of 210 cases. Int J Dermatol.

[8] Slominski A (2009). Neuroendocrine activity of the melanocyte. Exp Dermatol.

[9] Feldmeyer L, Keller M, Niklaus G, Hohl D, Werner S, Beer HD (2007). The inflammasome mediates UVB-induced activation and secretion of interleukin-1β by keratinocytes. Curr Biol.

[10] Watanabe H, Gaide O, Pétrilli V, Martinon F, Contassot E, Roques S, Kummer JA, Tschopp J, French LE (2007). Activation of the IL-1β-Processing inflammasome is involved in contact hypersensitivity. J Invest Dermatol.

[11] Wang CQ, Cruz-Inigo AE, Fuentes-Duculan J, Moussai D, Gulati N, Sullivan-Whalen M, Gilleaudeau P, Cohen JA, Krueger JG (2011). Th17 cells and activated sendritic cells are increased in vitiligo lesions. PLoS One.

[12] Laddha NC, Dwivedi M, Mansuri MS, Singh M, Patel HH, Agarwal N, Shah AM, Begum R (2014). Association of neuropeptide Y (NPY), interleukin-1B (IL1B) genetic variants and correlation of IL1B transcript levels with vitiligo susceptibility. PLoS One.

[13] Renne J, Schäfer V, Werfel T, Wittmann M (2010). Interleukin-1 from epithelial cells fosters T cell-dependent skin inflammation. Br J Dermatol.

[14] Cui D, Zhong F, Lin J, Wu Y, Long Q, Yang X, Zhu Q, Huang L, Mao Q, Huo Z, Zhou Z, Xie G, Zheng S (2017). Changes of circulating Th22 cells in children with hand, foot, and mouth disease caused by enterovirus 71 infection. Oncotarget.

[15] Dudakov JA, Hanash AM, van den Brink MRM (2015). Interleukin-22: immunobiology and pathology. Annu Rev Immunol.

[16] Nograles KE, Zaba LC, Shemer A, Fuentes-Duculan J, Cardinale I, Kikuchi T, Ramon M, Bergman R, Krueger JG, Guttman-Yassky E (2009). IL-22-producing “T22” T cells account for upregulated IL-22 in atopic dermatitis despite reduced IL-17-producing TH17 T cells. J Allergy Clin Immunol.

[17] Nograles KE, Zaba LC, Guttman E, Fuentes-Duculan J, Suarez-Farinas M, Cardinale I, Khatcherian A, Gonzalez J, Pierson KC, White TR, Pensabene C, Coats I, Novitskaya I (2008). Th17 cytokines interleukin (IL)-17 and IL-22 modulate distinct inflammatory and keratinocyte-response pathways. Br J Dermatol.

[18] Xie MH, Aggarwal S, Ho WH, Foster J, Zhang Z, Stinson J, Wood WI, Goddard AD, Gurney AL (2000). Interleukin (IL)-22, a novel human cytokine that signals through the interferon receptor-related proteins CRF2–4 and IL-22R. J Biol Chem.

[19] Dumoutier L, Van Roost E, Colau D, Renauld JC (2000). Human interleukin-10-related T cell-derived inducible factor: molecular cloning and functional characterization as an hepatocyte-stimulating factor. Proc Natl Acad Sci USA.

[20] Kotenko SV, Izotova LS, Mirochnitchenko OV, Esterova E, Dickensheets H, Donnelly RP, Pestka S (2001). Identification of the functional interleukin-22 (IL-22) receptor complex: the IL-10R2 chain (IL-10Rbeta) is a common chain of both the IL-10 and IL-22 (IL-10-related T cell-derived inducible factor, IL-TIF) receptor complexes. J Biol Chem.

[21] Blumberg H, Conklin D, Xu W, Grossmann A, Brender T, Carollo S, Eagan M, Foster D, Haldeman BA, Hammond A, Haugen H, Jelinek L, Kelly JD (2001). Interleukin 20: discovery, receptor identification, and role in epidermal function. Cell.

[22] Rutz S, Wang X, Ouyang W (2014). The IL-20 subfamily of cytokines from host defence to tissue homeostasis. Nat Rev Immunol.

[23] Aggarwal S, Xie MH, Maruoka M, Foster J, Gurney AL (2001). Acinar cells of the pancreas are a target of interleukin-22. J Interferon Cytokine Res.

[24] Wolk K, Kunz S, Witte E, Friedrich M, Asadullah K, Sabat R (2004). IL-22 increases the innate immunity of tissues. Immunity.

[25] Rutz S, Eidenschenk C, Ouyang W (2013). IL-22, not simply a Th17 cytokine. Immunol Rev.

[26] Nagalakshmi ML, Rascle A, Zurawski S, Menon S, de Waal Malefyt R (2004). Interleukin-22 activates STAT3 and induces IL-10 by colon epithelial cells. Int Immunopharmacol.

[27] Lejeune D, Dumoutier L, Constantinescu S, Kruijer W, Schuringa JJ, Renauld JC (2002). Interleukin-22 (IL-22) activates the JAK/STAT, ERK, JNK, and p38 MAP kinase pathways in a rat hepatoma cell line. Pathways that are shared with and distinct from IL-10. J Biol Chem.

[28] Gröne A (2002). Keratinocytes and cytokines. Vet Immunol Immunopathol.

[29] Dong C (2008). TH17 cells in development: an updated view of their molecular identity and genetic programming. Nat Rev Immunol.

[30] Seiberg M, Paine C, Sharlow E, Andrade-Gordon P, Costanzo M, Eisinger M, Shapiro SS (2000). The protease-activated receptor 2 regulates pigmentation via keratinocyte-melanocyte interactions. Exp Cell Res.

[31] Jordens I, Westbroek W, Marsman M, Rocha N, Mommaas M, Huizing M, Lambert J, Naeyaert JM, Neefjes J (2006). Rab7 and Rab27a control two motor protein activities involved in melanosomal transport. Pigment Cell Res.

[32] Beaumont KA, Hamilton NA, Moores MT, Brown DL, Ohbayashi N, Cairncross O, Cook AL, Smith AG, Misaki R, Fukuda M, Taguchi T, Sturm RA, Stow JL (2011). The recycling endosome protein Rab17 regulates melanocytic filopodia formation and melanosome trafficking. Traffic.

[33] Weiner L, Fu W, Chirico WJ, Brissette JL (2014). Skin as a living coloring book: how epithelial cells create patterns of pigmentation. Pigment Cell Melanoma Res.

[34] Van Den Bossche K, Naeyaert JM, Lambert J (2006). The quest for the mechanism of melanin transfer. Traffic.

[35] Yamaguchi Y, Hearing VJ (2009). Physiological factors that regulate skin pigmentation. BioFactors.

[36] Bucci C, Thomsen P, Nicoziani P, McCarthy J, van Deurs B (2000). Rab7: a key to lysosome biogenesis. Mol Biol Cell.

[37] Cantalupo G, Alifano P, Roberti V, Bruni CB, Bucci C (2001). Rab-interacting lysosomal protein (RILP): the Rab7 effector required for transport to lysosomes. EMBO J.

[38] Fukuda M (2013). Rab27 effectors, pleiotropic regulators in secretory pathways. Traffic.

[39] Strom M, Hume AN, Tarafder AK, Barkagianni E, Seabra MC (2002). A family of Rab27-binding proteins. Melanophilin links Rab27a and myosin Va function in melanosome transport. J Biol Chem.

[40] Wu X, Wang F, Rao K, Sellers JR, Hammer JA (2002). Rab27a is an essential component of melanosome receptor for myosin Va. Mol Biol Cell.

[41] Kameyama K, Sakai C, Kuge S, Nishiyama S, Tomita Y, Ito S, Wakamatsu K, Hearing VJ (1995). The expression of tyrosinase, tyrosinase-related proteins 1 and 2 (TRP1 and TRP2), the silver protein, and amelanogenic inhibitor in human melanoma cells of differing melanogenic activities. Pigment Cell Res.

[42] Fang D, Kute T, Setaluri V (2001). Regulation of tyrosinase-related protein-2 (TYRP2) in human melanocytes: relationship to growth and morphology. Pigment Cell Res.

[43] Park SH, Kim DS, Kim WG, Ryoo IJ, Lee DH, Huh CH, Youn SW, Yoo ID, Park KC (2004). Terrein: a new melanogenesis inhibitor and its mechanism. Cell Mol Life Sci.

[44] Ripoll VM, Meadows NA, Raggatt LJ, Chang MK, Pettit AR, Cassady AI, Hume DA (2008). Microphthalmia transcription factor regulates the expression of the novel osteoclast factor GPNMB. Gene.

[45] Loftus SK, Antonellis A, Matera I, Renaud G, Baxter LL, Reid D, Wolfsberg TG, Chen Y, Wang C, Prasad MK, Bessling SL, McCallion AS, NISC Comparative Sequencing Program (2009). Gpnmb is a melanoblast-expressed, MITF-dependent gene. Pigment Cell Melanoma Res.

[46] Taïeb A (2011). Vitiligo as an inflammatory skin disorder: a therapeutic perspective. Pigment Cell Melanoma Res.

[47] Marie J, Kovacs D, Pain C, Jouary T, Cota C, Vergier B, Picardo M, Taieb A, Ezzedine K, Cario-André M (2014). Inflammasome activation and vitiligo/nonsegmental vitiligo progression. Br J Dermatol.

[48] Wolk K, Haugen HS, Xu W, Witte E, Waggie K, Anderson M, Baur E, Witte K, Warszawska K, Philipp S, Johnson-Leger C, Volk HD, Sterry W, Sabat R (2009). IL-22 and IL-20 are key mediators of the epidermal alterations in psoriasis while IL-17 and IFN-γ are not. J Mol Med.

[49] Kim NS, Cho JH, Kang WH (2000). Behavioral differences between donor site-matched adult and neonatal melanocytes in culture. Arch Dermatol Res.

[50] Tomita Y, Maeda K, Tagami H (1992). Melanocyte-stimulating properties of arachidonic acid metabolites: possible role in postinflammatory pigmentation. Pigment Cell Res.

[51] Zhou J, Shang J, Song J, Ping F (2013). Interleukin-18 augments growth ability of primary human melanocytes by PTEN inactivation through the AKT/NF-κB pathway. Int J Biochem Cell Biol.

